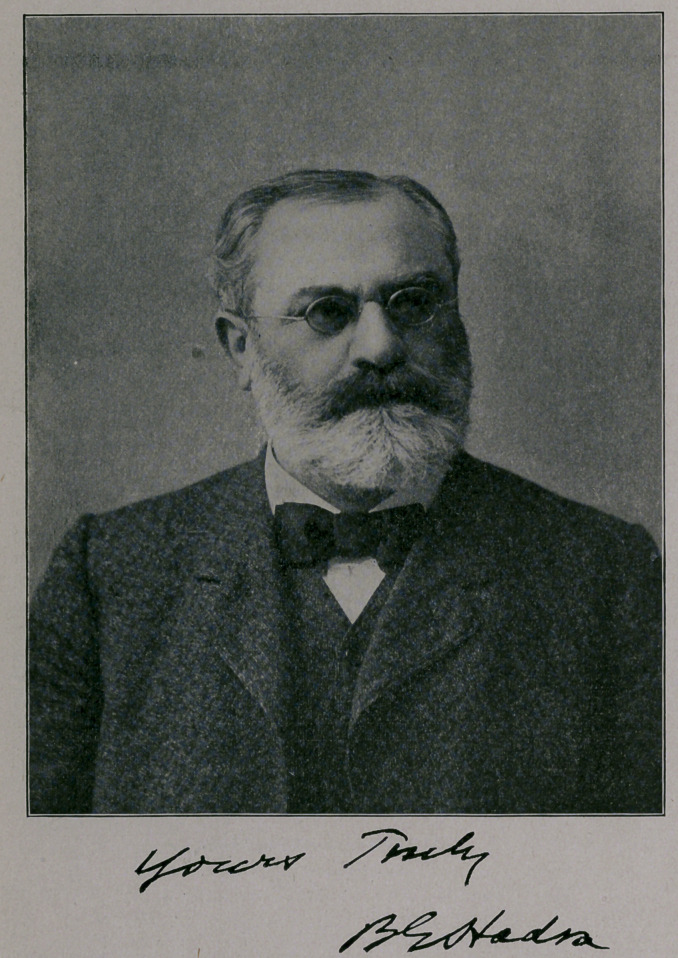# Dr. B. E. Hadra, President of the Texas State Medical Association

**Published:** 1900-06

**Authors:** 


### DR. B. E. HADRA,

### PRESIDENT OF THE TEXAS STATE MEDICAL ASSOCIATION.

---

BERTHOLD ERNEST HADRA, born in 1842 in Germany, received his medical education in the universities of Breslau and Berlin, from which latter he graduated and where he passed his state examination.

He served as volunteer surgeon in the war against Austria (1866), and afterwards entered the Prussian army service.

In 1870 he immigrated to Texas, where he has since resided. He practiced his profession in Austin, Galveston and San Antonio. He was a member of the board of regents of the University of Texas; held the chair of surgery in the old Texas Medical College, and was health officer of San Antonio. His contributions to medical literature are numerous. Aside from a monograph on "Injuries of the Pelvic Floor," he was the first one to devise conservative surgical treatment in place of oophorectomy, the so-called liberation of the pelvic organs. He was also the first one to propose total eventration of the contents and thorough washing and draining of the abdominal cavity in diffuse peritonitis. Repair of cystocele, perineum, etc., were frequent subjects of papers. To the surgery of the spine he contributed by adding wiring of the vertebræ. He has written also on the surgical treatment of epilepsy. To these many other original contributions, frequently quoted in international literature, may be added, such, for instance, as his paper on the open treatment of torticollis, on non-malignant tumors of the omentum, on relapsing appendicitis, on intestinal and gastric operations, etc.

We have the pleasure of presenting herewith an excellent picture of Dr. Hadra.

## Figures and Tables

**Figure f1:**